# Are Medical Missions a Nuisance?

**Published:** 1905-12-23

**Authors:** 


### ARE MEDICAL MISSIONS A NUISANCE?

At Bethnal Green on Thursday last there was an inquest on the body of an infant of five weeks old. It appeared from the evidence that the child had been taken by its mother to the Home of Industry Medical Mission to have its cold cured. When she arrived she found that one of the two doctors was ill, and after waiting for an hour she was informed that the other had telegraphed that he, too, was unwell. She had therefore to go away without advice, and the child subsequently died from bronchitis accelerated by fog. The coroner, Dr. Wynn Westcott, commenting on the case, said that these medical missions caused considerable trouble. His experience is that they treat people for minor ailments, but that if the patient is taken worse, there is no one to attend; and his opinion is "that any medical charity which does not have a medical man on the premises is a nuisance." This seems to us a sweeping pronouncement. We quite admit that if there are medical missions at which advice is given to poor people by individuals who have no professional qualifications at all, they ought to be promptly suppressed. But at Bethnal Green no attempt was made by an unauthorised person to prescribe, and though the simultaneous absence of the two medical men was very unfortunate, we are not prepared to say that, because a child dies from bronchitis, naturally augmented by a journey through the fog, blame of necessity attaches to the mission. It is probable that if the mother had kept the baby warm at home it would have had a better chance of surviving than if it had been immediately attended to by a qualified doctor, but still had to face the effects of weather, which is particularly disastrous to young and delicate life. If all medical missions are to be condemned as nuisances unless there be a doctor always on the premises, what about cottage hospitals and many other institutions which, whatever their individual defects, are often of great public service?

